# Clinical Outcomes of Robotic Versus Laparoscopic Colorectal Surgery During the Early Learning Curve: A Systematic Review and Meta-Analysis

**DOI:** 10.7759/cureus.86196

**Published:** 2025-06-17

**Authors:** Masood U Rehman, Reem Moussa, Naeem Ahmed, Arjuna Brodie, Talha Tarrar, Abdul Rehman, Kamran Malik, Jamil Ahmed

**Affiliations:** 1 General Surgery, Northampton General Hospital, Northamptonshire, GBR

**Keywords:** laparoscopic, learning curve, outcomes, robotic, robotic-assisted

## Abstract

Surgery is a cornerstone for colorectal cancer treatment. Though laparoscopy surgery is a well-established technique for colorectal patients and has shown reduced hospital stay and postoperative morbidities, it has inherent limitations due to straight instruments and limited views in areas such as the pelvis. Nevertheless, recently, the robotic approach has aimed to overcome the limitations of laparoscopic techniques and offers EndoWrist technology with 3D, high definition, and stable views for precise dissection.

This review aims to compare the clinical outcomes of laparoscopic and robotic surgery for colorectal cancer during the initial adoption phase of robotic techniques.

A comprehensive literature search was conducted according to the Preferred Reporting Items for Systematic Reviews (PRISMA) guidelines, resulting in the inclusion of six randomized controlled trials (RCTs) and 17 observational studies. Eligibility criteria focused on adult patients undergoing elective resection for colorectal neoplasia.

A total of 23 articles were analyzed for various outcomes. Robotic surgery demonstrated a shorter postoperative hospital stay compared to laparoscopic surgery (MD = −0.132; p = 0.031) and significantly lower conversion to open surgery (OR = 1.480; 95% CI: 0.364-0.635; p = 0.000). No significant difference was observed in 30-day mortality, early postoperative complications, readmission, and reoperation rates between the two groups.

Emerging evidence suggests that, during the early adoption phase, robotic surgery for colorectal cancer is associated with reduced conversion rates to open surgery and shorter postoperative hospital stays compared to laparoscopic surgery. However, comprehensive evaluation through future studies is required to elucidate long-term outcomes, cost-effectiveness, and patient-reported measures related to robotic colorectal surgery.

## Introduction and background

Colorectal cancer is ranked as the third most prevalent cancer globally and affects both men and women equally. It is the fourth most common cancer in the United Kingdom (UK), with approximately 44,000 new cases diagnosed annually (Bowel Cancer UK) [1]. In the UK, colorectal cancer accounts for around 11% of all new cancer cases each year, making it a major public health concern [2]. Despite advancements in screening programs such as the NHS Bowel Cancer Screening Programme, a significant proportion of cases are still diagnosed at an advanced stage, contributing to considerable morbidity and mortality. The incidence of colorectal cancer is notably higher among individuals over the age of 50, although an increasing trend is being observed in younger age groups as well [3].

During the last decade, the diagnostics, staging, and treatment options for colorectal cancers have improved and have resulted in better outcomes for patients [4-6]. These outcomes are based on numerous clinical trials and research initiatives, adopting the latest surgical technology [7-10]. Regular screening has become a cornerstone in the fight against colorectal cancer, leading to a 90% five-year survival rate when the disease is detected at an early stage [2]. Unfortunately, despite screening programs, a substantial number of cases of colorectal cancer are undetected until later stages, thus intensifying the focus on improving treatment approaches [3]. In contrast, a worrying 2-3% annual increase in colorectal cancer incidence has been reported among individuals under the age of 50, underscoring the urgent need for continued clinical research [1].

The management of colorectal cancer involves a multimodal approach, including surgery, chemotherapy, radiotherapy, or a combination of these modalities. Among them, surgery remains the cornerstone of treatment [11-13]. Surgical techniques may be performed via open or minimally invasive methods, including laparoscopic and robotic approaches [14-16]. Notably, laparoscopic surgery is a well-established, evidence-based technique for colorectal resections, offering advantages such as reduced postoperative pain, lower morbidity, faster recovery, and improved quality of life [17,18].

Robotic surgery has rapidly gained recognition as a viable and safe minimally invasive alternative for colorectal procedures, building on technological advancements originally developed for aerospace, engineering, and automotive industries [19-29,30]. By offering enhanced instrument articulation, 3D visualization, and improved ergonomics, the robotic approach seeks to overcome the intrinsic limitations of conventional laparoscopy [30-35]. However, despite promising early results, comparative evidence between robotic and laparoscopic techniques remains inconclusive, particularly regarding short-term clinical outcomes. Major studies, including the Robotic Versus Laparoscopic Surgery for Middle and Low Rectal Cancer (REAL) trial and the Robotic Versus Laparoscopic Resection for Rectal Cancer (ROLARR) trial, have reported inconsistent findings, underscoring the need for further high-quality analyses [33-35]. In this context, our study provides a timely and comprehensive evaluation of surgical outcomes during the initial adoption phase of robotic colorectal surgery [32,33]. 

This systematic review and meta-analysis endeavors to compare early surgical outcomes between laparoscopic and robotic surgery, ultimately determining whether robotic surgery offers better or equal clinical outcomes even at the early stage of the adaptation of the robotic approach.

## Review

Materials and methods 

This meta-analysis was performed according to the guidelines of the Preferred Reporting Items for Systematic Reviews (PRISMA) [7]. 

Literature Search

A thorough literature search was conducted across MEDLINE (via PubMed), Web of Science, the Cochrane Library, and ClinicalTrials.gov to identify relevant studies from inception through July 2022. Additionally, the reference lists of the retrieved articles were reviewed to uncover further pertinent studies. The search strategy included the following terms: “colorectal surgery” OR “colorectal procedures” OR “colon surgery” OR “rectal surgery” OR “early postoperative complications” OR “postoperative complications within 30 days” OR “perioperative complications” OR “postoperative recovery” OR “recovery of function” OR “patient recovery” OR “conversion rate” OR “conversion to open surgery” OR “perioperative mortality” OR “surgical mortality” OR “death during surgery” OR “length of hospital stay” OR “hospitalization duration” OR “length of stay” OR “unscheduled reoperation” OR “reoperation within 30 days” OR “repeat surgery within 30 days.” This study is registered with PROSPERO under the registration number CRD42023468608.

Eligibility Criteria

The eligibility criteria followed the population, intervention, comparators, study design, and outcome (PICOS) strategy:

Population: Adult patients with rectal/colorectal or colon neoplasia of any stage (I-IV) undergoing resection.

Intervention: Robot-assisted resection.

Comparator: Laparoscopic resection.

Study design: Randomized controlled trials (RCTs) and observational studies that compared the two surgical techniques were included.

Outcome: Studies that reported early clinical outcomes after adopting the robotic approach were included. The outcomes included postoperative complications (pain, anastomotic leak, bleeding, and sepsis), reoperation, length of hospital stay, up to 90-day mortality, conversion to open surgery, readmission, and reoperation. 

Studies were excluded if they reported insufficient data, lacked relevant outcomes, focused solely on long-term follow-up, were review articles, involved cadaveric or animal models, or presented overlapping data from the same patient population.

Study Selection and Data Extraction

All titles and abstracts were screened for eligibility based on the predefined inclusion and exclusion criteria. Full texts of potentially relevant articles were then reviewed in detail by two independent investigators. Data from eligible studies were extracted into a standardized Microsoft Excel spreadsheet (version 2019, Microsoft). Any discrepancies were resolved through discussion with a third reviewer. The extracted data included: first author, year of publication, country of origin, study design, patient age, gender, body mass index (BMI, kg/m²), and outcomes of interest.

Quality Assessment

The quality of the included studies was assessed using the Cochrane Risk of Bias 2 (RoB2) tool for RCTs and the Newcastle-Ottawa Scale (NOS) for observational studies [9]. Two independent reviewers conducted the quality assessments, and any disagreements were resolved through discussion with a third author.

Statistical Analysis

Data analysis was performed using Comprehensive Meta-Analysis (CMA) version 3.0. Dichotomous data are presented as odds ratios (ORs) and continuous data as mean differences (MDs). A random-effects model was used to account for the heterogeneity of included studies. An I2 index >75% is demonstrated as high heterogeneity. A p-value of <0.05 was considered statistically significant in all analyses.

Results 

Literature Search

**Figure 1:**
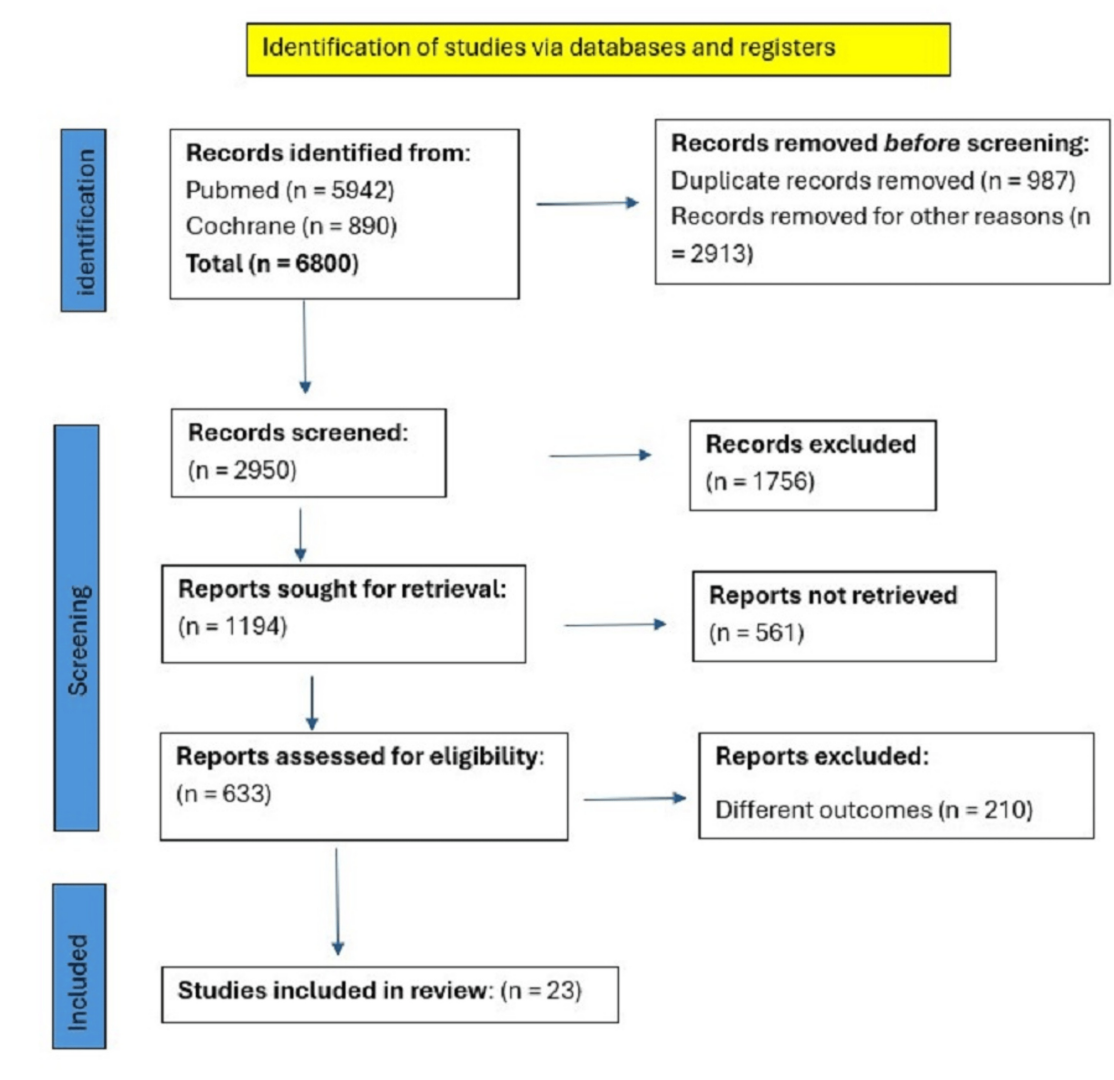
Preferred Reporting Items for Systematic Reviews and Meta-Analyses (PRISMA) flowchart of literature search

A total of 6850 articles were retrieved. After removing duplicates, 2950 articles were screened via titles and abstracts. Ultimately, 78 articles were selected for in-depth review. Finally, six RCTs [10-15] and 17 observational studies [16-32] were included in the final qualitative and quantitative meta-analysis. This selection process is illustrated in the PRISMA flowchart (Figure 1).

Study Characteristics

The characteristics of the included studies are summarized in Table 1. A total of 47,145 patients were analyzed in this meta-analysis, comprising 4388 patients in the robotic surgery group and 42,757 in the laparoscopic group. The studies were conducted across 11 different countries [10-32].

**Table 1:** Study characteristics RCT, randomized controlled trial; ASA, American Society of Anesthesiologists; N/A, not available; BMI, body mass index; USA, Unites States of America; UK, United Kingdom; I, intervention group; C, control group

Study	Design	Country	Sample size	Demographics	Population description	Outcomes
Xu et al., 2022 [10]	RCT	China	I = 586; C: 585	Age: I = 59.1 (11.0); C = 60.7 (9.8)	Middle or low rectal cancer ≤10 cm from anal verge	30-day mortality; conversion to open surgery; postoperative complications; length of hospital stay; readmission; reoperation
				Male/female: I = 356/230; C: 354/231		
				BMI: I = 23.5 (3.3); C = 23.5 (3.1)		
Kim et al., 2018 [11]	RCT	Korea	I = 66; C = 73	Age: I = 60.4 ± 9.7; C = 59.7 ± 11.7	Rectal adenocarcinoma ≤9 cm from anal verge, no metastasis	Postoperative complications; length of hospital stay
				Male/female: I = 51/15; C = 52/21		
				BMI: I = 24.1 ± 3.3; C = 23.6 ± 3.0		
Baik et al., 2008 [12]	RCT	Korea	I = 18; C = 18	Age: I = 57.3 ± 6.3; C = 62.0 ± 9.0	T1-3, M0 rectal cancer	Conversion to open surgery; length of hospital stay
				Male/female: I = 14/4; C = 14/4		
				BMI: I = 22.8 ± 1.8; C = 24.0 ± 2.5		
Debakey et al., 2018 [13]	RCT	Egypt	I = 21; C = 24	Age: I = 53.4 (32-67); C = 50.3 (36-64)	Rectal adenocarcinoma ≤15 cm from anal verge	Conversion to open surgery; length of hospital stay; readmission; reoperation
				Male/female: I = 11/10; C = 13/11		
				BMI: not specified		
Jayne et al., 2017 [14]	RCT	UK	I = 237; C = 234	Age: I = 64.4 ± 10.98; C = 65.5 ± 11.93	Rectal adenocarcinoma suitable for curative resection	30-day mortality; postoperative complications
				Male/female: I = 161/76; C = 159/75		
				Patriti et al., 2009 [15]			RCT	Italy	I = 29; C = 37	Age: I = 68 ± 10; C = 69 ± 10	Histologically proven rectal adenocarcinoma, any stage	30-day mortality; conversion to open surgery; length of hospital stay
Male/female: I = 11/18; C = 12/25												
BMI: I = 24 ± 6.2; C = 25.4 ± 6.44												
Ahmed et al., 2017 [16]	Prospective cohort	UK	I = 99; C = 85	Age: I = 69 (63-75); C = 68 (62-74)	History of chemoradiotherapy, tumor <8 cm from anal verge	Conversion to open surgery; length of hospital stay; readmission; reoperation
				Male/female: I = 71/21; C = 58/57		
				BMI: I = 27 (24-30); C = 27 (24-31)		
Mirkin et al., 2018 [17]	Retrospective cohort	USA	I = 765; C = 14,347	Age: not specified	Pathological stage I–III colon adenocarcinoma (ICDO-3: 8140/3)	Conversion to open surgery
				Male/female: I = 364/401; C = 7059/7288		
				BMI: not specified		
Yamaguchi et al., 2016 [18]	Prospective cohort	Japan	I = 203; C = 239	Age: I = 64.8 ± 10.8; C = 65.9 ± 10.8	Lower rectal cancer T3–4 or T1–2 with lateral lymph node metastasis	Conversion to open surgery; length of hospital stay
				Male/female: I = 140/63; C = 154/85		
				BMI: I = 23.4 ± 3.16; C = 23.1 ± 3.64		
Gorgun et al., 2016 [19]	Retrospective cohort	USA	I = 29; C = 27	Age: I = 58.8 ± 10.7; C = 60.3 ± 9.8	cT3-T4 or cN1-2 rectal cancer, neoadjuvant chemoradiation, surgery after 8-10 weeks	Conversion to open surgery; postoperative complications
				Male/female: I = 22/7; C = 16/11		
				BMI: I = 34.9 ± 7.2; C = 35.2 ± 5.0		
Hu et al., 2020 [20]	Retrospective cohort	USA	I = 1164; C = 2681	Age: I = 56.5 ± 15; C = 53.4 ± 16	Patients >18 yrs, elective surgeries, ASA class 1–4	Conversion to open surgery; length of hospital stay; readmission; reoperation
				Male/female: I = 688/476; C = 1427/1254		
				BMI: I = 28.0 ± 6; C = 27.2 ± 6		
Saklani et al., 2013 [21]	Prospective cohort	South Korea	I = 74; C = 64	Age: I = 59.6 ± 12.3; C = 60.1 ± 10.8	Mid and low rectal adenocarcinomas (0–12 cm from anal verge)	Postoperative complications; length of hospital stay; readmission; reoperation
				Male/female: I = 50/24; C = 46/18		
				BMI: I = 23.4 ± 2.9; C = 22.7 ± 2.9		
Feroci et al., 2016 [22]	Retrospective cohort	Italy	I = 53; C = 58	Age: I = 66 (33-80); C = 66 (42-84)	Middle and low rectal adenocarcinoma (≤12 cm from anal verge)	Conversion to open surgery; length of hospital stay
				Male/female: I = 27/26; C = 42/16		
				BMI: I = 24.6 (18-31); C = 24.6 (19-37)		
Garfinkle et al., 2019 [23]	Prospective cohort	Canada	I = 154; C = 213	Age: I = 61.9 ± 13.5; C = 63.8 ± 13.3	Elective proctectomy for rectal cancer (CPT codes)	30-day mortality; conversion to open; readmission; reoperation
				Male/female: I = 106/48; C = 127/86		
				BMI: I = 28.0 ± 6.1; C = 27.3 ± 5.8		
Del Gutiérrez Delgado et al., 2022 [24]	Retrospective cohort	Spain	I = 178; C = 122	Age: I = 66.1 ± 10.8; C = 67.6 ± 14.3	Rectal adenocarcinoma <15 cm from anal margin	Length of hospital stay; readmission; reoperation
				Male/female: I = 124/54; C = 67/55		
				BMI: I = 27.4 ± 3.9; C = 27.6 ± 4.8		
Cho et al., 2015 [25]	Retrospective cohort	South Korea	I = 278; C = 278	Age: I = 57.4 ± 11.6; C = 58.3 ± 10.4	Colorectal cancer, tumor <15 cm from anal verge	Conversion to open surgery; length of hospital stay
				Male/female: I = 182/96; C = 184/94		
				BMI: I = 23.5 ± 2.9; C = 23.7 ± 3.3		
de’Angelis et al., 2016 [26]	Prospective cohort	France	I = 30; C = 50	Age: I = 71 ± 8.5; C = 71.1 ± 12.92	Right colon cancer requiring surgical resection	30-day mortality; conversion to open surgery; length of hospital stay
				Male/female: I = 15/15; C = 19/13		
				BMI: I = 26.43 ± 3.21; C = 25.26 ± 4.19		
de’Angelis et al., 2018 [27]	Prospective cohort	France	I = 43; C = 43	Age: not specified	Solitary adenocarcinoma (colon or rectum), AJCC stage I–IVa	Length of hospital stay; reoperation
				Male/female: I = 23/20; C = 25/18		
				BMI: not specified		
Morelli et al., 2016 [28]	Retrospective cohort	Italy	I = 50; C = 25	Age: I = 68.8 ± 10.7; C = 68.9 ± 11.5	75 patients with rectal cancer	30-day mortality; length of hospital stay
				Male/female: I = 33/17; C = 15/10		
				BMI: I = 24.7 ± 3.5; C = 24.3 ± 4.2		
Fleming et al., 2021 [29]	Prospective cohort	UK	I = 64; C = 64	Age: I = 67 (12); C = 66 (13)	Elective laparoscopic/robotic resection for colorectal cancer (2016–2019)	Conversion to open surgery; readmission; reoperation
				Male/female: I = 37/27; C = 33/31		
				BMI: not specified		
Baek et al., 2012 [30]	Prospective cohort	Korea	I = 154; C = 150	Age: I = 59.1 ± 12.2; C = 62.3 ± 10.9	Primary rectal cancer ≤15 cm from anal verge	Postoperative complications; length of hospital stay
				Male/female: I = 105/49; C = 109/41		
				BMI: I = 23.4 ± 3.1; C = 23.1 ± 3.0		
Silva-Velazco et al., 2017 [31]	Prospective cohort	USA	I = 66; C = 118	Age: I = 59 (29-77); C = 60 (30-89)	Primary rectal cancer, curative intent proctectomy	Conversion to open surgery; readmission; reoperation
				Male/female: I = 50/16; C = 66/52		
				BMI: I = 29.5 (22-66); C = 27 (16-45)		
Chen and Liang, 2022 [32]	Prospective cohort	Taiwan	I = 56; C = 56	Age: I = 57.4 ± 11.2; C = 56.3 ± 11.0	N/A	30-day mortality; postoperative complications; length of hospital stay
				Male/female: I = 39/38; C = 38/18		
				BMI: not specified		

Risk of Bias Assessment

According to the RoB2 tool, four out of the six RCTs adequately described the method used for random sequence generation [10-12,14]. Consequently, these four studies were considered to have a low risk of bias. In contrast, one study was rated as having an unclear risk of bias due to insufficient information regarding the randomization process [13], while one was deemed high-risk [15]. Random allocation concealment was adequately reported in four studies, and these were consequently assessed as having a low risk of bias in this domain [10-12,14]. Two studies exhibited certain methodological concerns regarding allocation concealment and were therefore assessed as having a high risk of bias in this domain [13,14]. Most of the included studies did not sufficiently report on blinding of participants, personnel, or outcome assessment and were therefore judged to have a high risk of bias in this domain. Notably, none of the studies reported missing outcome data. In terms of other potential sources of bias, all studies were assessed as having a low risk.

The quality assessment for observational studies, carried out by NOS, showed that only one study was given a score of 829, while scores of 7, 6, and 5 were given to a total of seven (41.41%), four (43.5%), and four studies (43.5%), respectively.

The quality assessment of the six RCTs and 17 non-randomized prospective studies is presented in Tables 2, 3 [10-15].

**Table 2:** Risk of bias for RCTs RCT, randomized controlled trial

Author (year)	Random sequence generation	Allocation concealment	Selective reporting	Blinding of participants/personnel	Blinding of outcome assessment	Incomplete outcome data	Other sources of bias
Xu et al., 2022 [10]	Low risk	Low risk	Low risk	High risk	Low risk	Low risk	Low risk
Kim et al., 2018 [11]	Low risk	Low risk	Low risk	High risk	High risk	Low risk	Low risk
Baik et al., 2008 [12]	Low risk	Low risk	Unclear	Low risk	Unclear	Low risk	Low risk
Debakey et al., 2018 [13]	Unclear	High risk	Low risk	High risk	High risk	Low risk	Low risk
Jayne et al., 2017 [14]	Low risk	Low risk	Low risk	Low risk	Low risk	Low risk	Low risk
Patriti et al., 2009 [15]	High risk	High risk	High risk	High risk	High risk	High risk	Low risk

**Table 3:** Newcastle-Ottawa Quality Assessment Scale for cohort studies

Study	Selection				Comparability	Outcome			Total
	1	2	3	4	5	6	7	8	
Ahmed et al., 2017 [16]	★	★	★	★	-	★	★	★	7
Mirkin et al., 2018 [17]	-	-	★	★	-	★	★	★	5
Yamaguchi et al., 2016 [18]	★	★	★	★	-		★	★	6
Gorgun et al., 2016 [19]	-	★	★	★	-	★	★	★	6
Hu et al., 2020 [20]	-	★	-	★	-	★	★	★	5
Saklani et al., 2013 [21]	★	-	★	★	★	★	★	★	7
Feroci et al., 2016 [22]	★	★	★	★	-	★	★	★	7
Garfinkle et al., 2019 [23]	★	★	-	★	-	★	★	★	6
Del Gutiérrez Delgado et al., 2022 [24]	★	★	★	★	-	★	★	★	7
Cho et al., 2015 [25]	★	★	★	★	-	★	★	★	7
de’Angelis et al., 2016 [26]	-	-	★	★	-	★	★	★	5
de’Angelis et al., 2018 [27]	-	-	★	★	-	★	★	★	5
Morelli et al., 2016 [28]	★	-	★	★	★	★	★	★	7
Fleming et al., 2021 [29]	★	★	★	★	★	★	★	★	8
Baek et al., 2012 [30]	★	★	★	★	-	★	★	★	7
Silva-Velazco et al., 2017 [31]	★	★	-	★	-	★	★	★	6
Chen and Liang, 2022 [32]	★	★	★	★	-	★	★	★	7

Publication Bias

**Figure 2:**
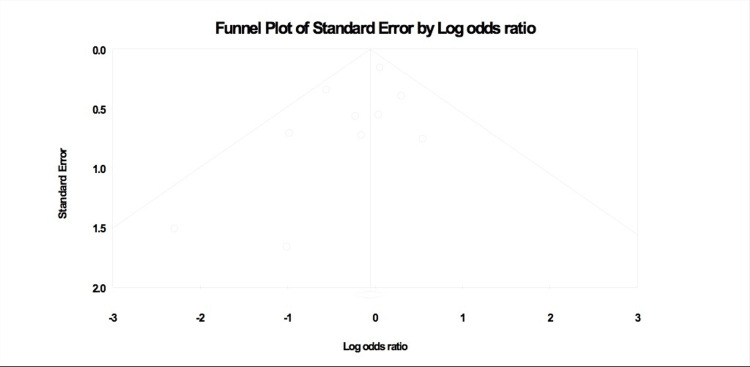
Funnel plot representing publication bias

The publication bias between studies is illustrated in Figure 2.

Quantitative Analysis

The final 23 articles were analyzed for our desired outcomes.

Thirty-day mortality: A total of seven out of 23 studies reported 30-day mortality in 2342 participants, of which 1142 belonged to the robotic arm and 1200 belonged to the laparoscopic surgery group [10,14,15,23,26,28,32]. Our analysis found that there was no statistical significance (OR = 1.061; 95% CI: 0.579-1.940; p = 0.85) between the groups. No heterogeneity was found between studies in this analysis (I2 = 0%; p = 0.85).

**Figure 3:**
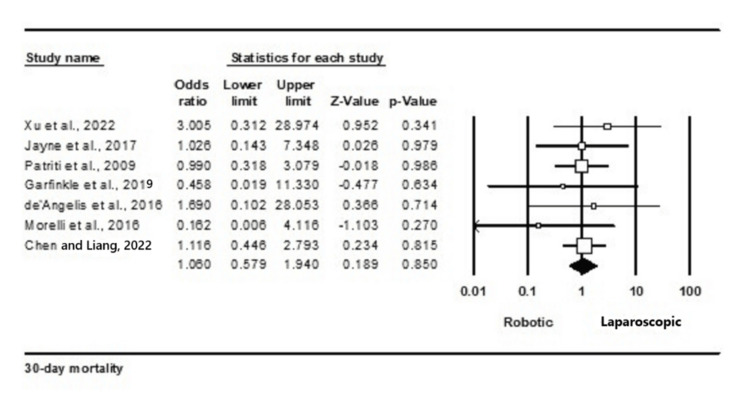
Forest plot for 30-day mortality

The outcome is illustrated in Figure 3.

**Figure 4:**
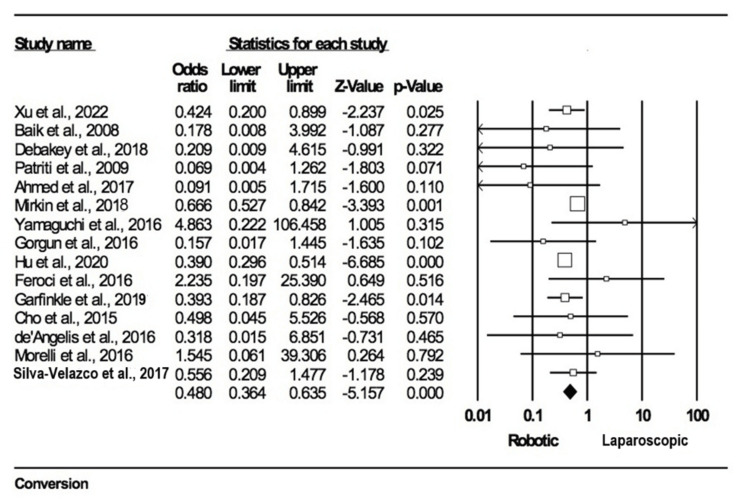
Forest plot for conversion to open surgery

Conversion to open surgery: Fifteen studies [10,11,13,15-20,22-24,26,28,31] reported conversion to open surgery as one of their outcomes, with total participation from 22,052 individuals: 3545 in the robotic group and 18,507 in the laparoscopic group. A significant statistical relationship was found, and our analysis revealed favorability toward the robotic arm (OR = 1.480; 95% CI: 0.364-0.635; p = 0.000). There was minimal heterogeneity observed across the studies (I2 = 23.5%, p = 0.193) (Figure 4).

**Figure 5:**
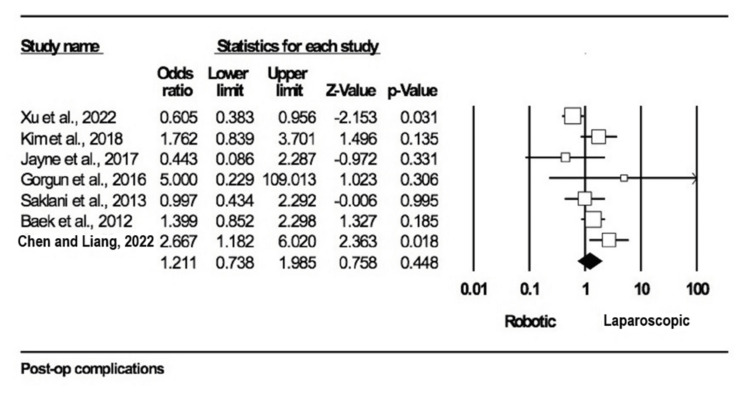
Forest plot for early post-operative complications

Early postoperative complications: A total of seven studies [10,11,14,19,21,30,32] included this outcome, with 2391 participants: 1202 in the robotic arm and 1189 in the laparoscopic one. There were no statistically significant differences in early postoperative complications between the open surgery and robotic surgery group (OR = 1.211; 95% CI: 0.738-1.985; p = 0.448), and evidence of heterogeneity was found among the included studies (I2 = 61%; p = 0.015) (Figure 5).

**Figure 6:**
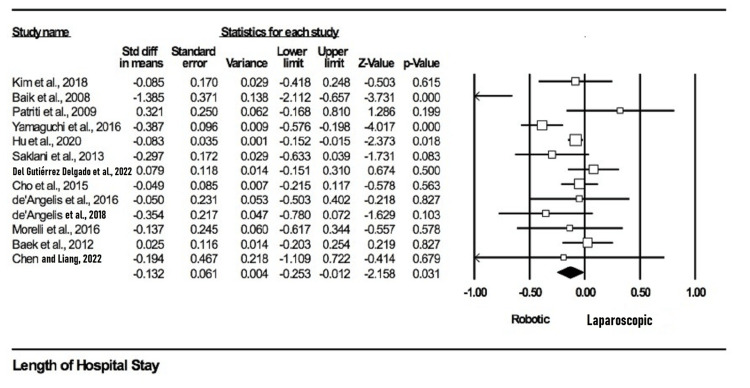
Forest plot of post-operative hospital stay

Postoperative hospital stay: Thirteen studies reported and compared postoperative stay between both groups [11,12,15,18,20,21,24-28,30,32]. Given that the outcome was continuous, the data were aggregated as MD. A total of 6235 participants were found in both groups, with 2343 in the robotic arm and 3836 in the laparoscopic arm. In the final pooled analysis, hospital stay was significantly shorter in the robotic surgery group compared to the open surgery group (MD = −0.132; p = 0.031). The meta-analysis showed moderate heterogeneity between studies (I2 = 60.74%; p = 0.002). The outcome is illustrated in Figure 6.

**Figure 7:**
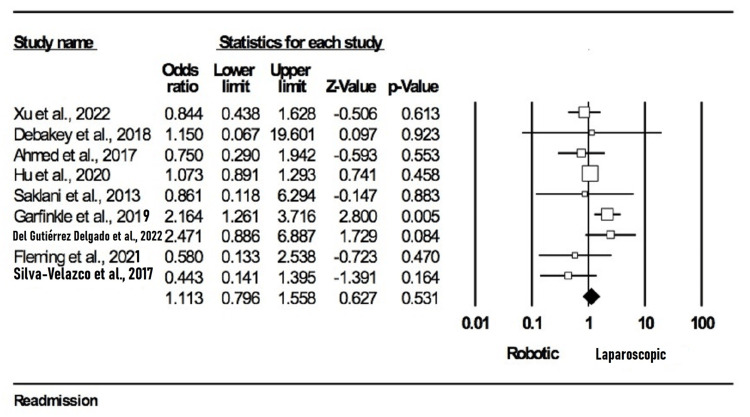
Forest plot for Readmission

Readmission: Nine studies [10,13,16,20,21,23,24,29,31] reported this outcome with a total of 6392 participants (2406 in the robotic group and 3956 in the laparoscopic group). Our analysis revealed that there was no statistical significance (OR = 1.113; 95% CI: 0.796-1.558; p = 0.53) between the groups when it came to readmission. We found small heterogeneity between studies during our analysis (I2 = 38.07%; p = 0.115). The outcome is illustrated in Figure 7.

**Figure 8:**
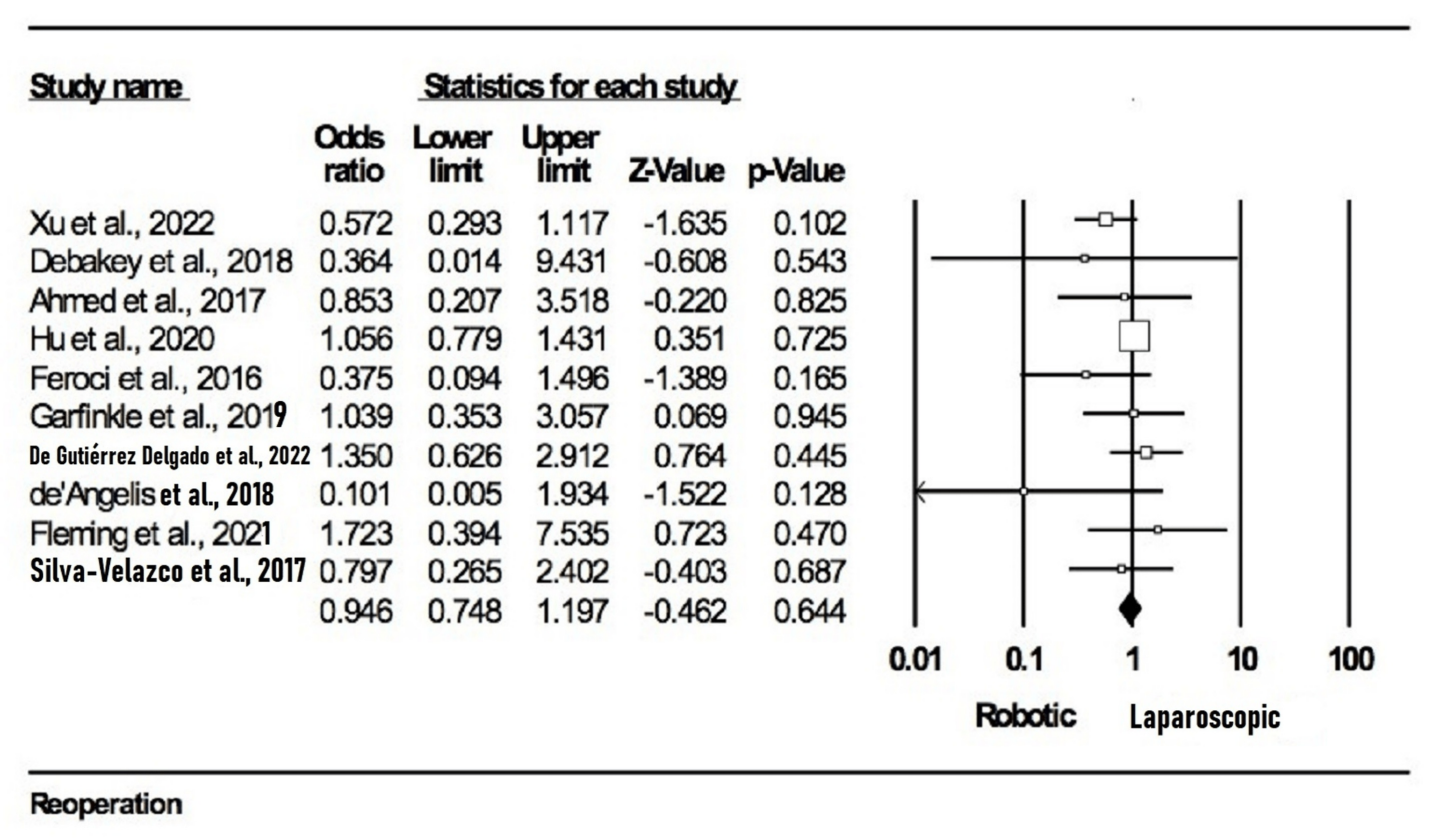
Forest plot for reoperation

Reoperation: This outcome aimed to assess the number of participants in each group who underwent a second procedure in the event that the first was deemed unsuccessful. In our meta-analysis, 10 studies [10,13,16,20,22-24,27,29,31] evaluated this outcome with a total of 6421 participants (2428 in the robotic group and 3993 in the laparoscopic group). There were no statistically significant differences in reoperation rates between the open surgery and robotic surgery group (OR = 0.946; 95% CI: 0.748-1.197; p = 0.644), and no evidence of heterogeneity was found among the included studies (I2 = 0%; p = 0.482) (Figure 8).

Surgeons’ experience: It is important to highlight the experience of the surgeons included in this review. Most surgeons had significant experience in laparoscopic colorectal surgery before starting to perform robotic surgery. This means they were skilled laparoscopic surgeons but were still early in their learning curve for robotic surgery. Despite being new to robotic techniques, the results showed that clinical outcomes were comparable, and sometimes even better, than laparoscopic surgery. This suggests that having a strong background in laparoscopic surgery, along with proper training in robotic surgery, helped surgeons achieve good outcomes even during the early adoption phase. It also highlights the value of structured training programs in supporting the safe and effective use of robotic surgery in colorectal practice.

Discussion 

In recent decades, minimally invasive techniques have garnered significant attention owing to their numerous benefits, including reduced blood loss, lower complication rates, and shorter hospital stays, among others [5]. In the management of colorectal carcinoma, robotic, laparoscopic, and open surgical approaches have remained the primary modalities for resection over the past several years [4]. Thereby, many investigators have worked to evaluate and compare minimal techniques with open surgery. However, we found that most studies evaluated other parameters such as long-term outcomes, cost discrepancy, oncological outcomes, or impact on quality of life; other studies gathered data from retrospective trials, RCTs, or prospective cohorts [14,33-36].

Our analysis focused on surgical outcomes between robotic and laparoscopic arms at the early stage of the adaptation of the robotic approach. Consequently, this analysis is possibly the most comprehensive to date in terms of the number of studies reviewed. It is believed that most of the surgeons, if not all, before embarking on the robotic approach had extensive laparoscopic experience. This makes them experienced surgeons for the laparoscopic approach but novices for the robotic approach [16,19,29].

Our primary outcome of interest, 30-day mortality, did not yield statistically significant results. This was in line with previous literature, which pointed out no discernible difference between the two groups [2]. Similarly, other outcomes, including readmission, reoperation, and early postoperative complications, signified that both groups are equally competitive [33].

Two of our outcomes (conversion to open surgery and length of hospital stay) revealed a statistically significant improvement in robotic surgery compared to laparoscopic surgery. This can be attributed to the fact that robotic surgeries offer better visualization in a narrow operative field, increased precision, and improved ergonomics for surgeons. This also, in general, led to better outcomes, which translated to shorter hospital stays. This finding contrasts with the literature, suggesting that while robotic arms do lead to shorter hospital stays compared to open surgeries, they do not do so when compared with laparoscopic ones [2]. Less conversion to open and shorter hospital duration have also often been associated with reduced complication rates, and while traditionally it is widely acclaimed that robotic surgery yields fewer complications, our analysis did not demonstrate that.

It is important to highlight the surgical experience of the teams included in this review. Most surgeons had substantial experience in laparoscopic colorectal surgery before transitioning to robotic surgery. As a result, while they were expert laparoscopic surgeons, they were relatively new to robotic techniques and still within their learning curve. Despite this, our analysis shows that outcomes with robotic surgery were comparable, and in some cases superior, to those with laparoscopy [27-30,32-34].

This likely reflects the advantage of having a strong foundation in minimally invasive surgery, supported by structured robotic training programs [35,36]. These training programs often include simulation-based practice, use of dual-console robotic systems for supervised learning, participation in proctored cases, and dedicated robotic surgery fellowships or certification courses. Such structured training enables surgeons to safely adopt robotic surgery and achieve good clinical outcomes even early in their robotic experience. This finding highlights the importance of effective training pathways when introducing new surgical technologies into clinical practice [37].

Given the positive outcomes observed even during the early learning curve, it is evident that structured and standardized training plays a crucial role in the successful adoption of robotic colorectal surgery [26]. Moving forward, the development of formal credentialing processes, proficiency-based training modules, and the widespread use of simulation and dual-console mentorship systems will be essential [23-25]. Establishing standardized robotic surgery curricula across centers may help ensure consistent surgical quality, minimize learning curve-related complications, and optimize patient outcomes. Future studies should also explore the impact of different training methods on surgical performance and long-term clinical results [16,23,27].

While robotic surgery has garnered substantial attention, our analysis revealed that only two out of six assessed outcomes showed a statistically significant advantage over laparoscopic surgery. Given that robotic procedures are associated with considerably higher costs, largely driven by the complexity of the technology and maintenance requirements, it is crucial to critically assess their overall value. Although certain benefits, such as lower conversion rates and shorter hospital stays, were observed, the broader question remains: does robotic surgery offer sufficient clinical or economic superiority to warrant its widespread adoption over the significantly more cost-effective laparoscopic approach? [36,37]

Moving forward, rigorous evaluation of cost-effectiveness, patient-centered outcomes, and long-term oncological results is needed. Future research should also focus on defining optimal patient selection criteria and refining technical strategies to maximize the benefits of robotic surgery while minimizing unnecessary healthcare expenditure [5,6,10,11].

This study has several limitations that should be acknowledged. First, there was considerable heterogeneity among the included studies, as the meta-analysis incorporated both RCTs and observational studies, introducing variability in patient populations, surgical techniques, and institutional practices. Second, the potential for publication bias exists, as studies with statistically significant findings are more likely to be published, possibly leading to an overestimation of treatment effects. Moreover, our analysis did not evaluate long-term outcomes such as recurrence rates, overall survival, or functional recovery, limiting conclusions regarding the long-term impact of robotic versus laparoscopic surgery [13,15,16,33-36].

In addition, confounding factors, including surgeon experience, patient selection criteria, and institutional preferences, may have influenced the reported outcomes. It is also notable that operative time, an important perioperative parameter, was not consistently reported across the included studies and therefore could not be analyzed. The inability to fully adjust for these confounding variables may introduce bias and affect the generalizability of the results. Future studies addressing these limitations are warranted to provide a more comprehensive understanding of the role of robotic surgery in colorectal cancer care.

## Conclusions

In conclusion, this meta-analysis evaluated early clinical outcomes of robotic versus laparoscopic colorectal cancer surgery during the initial phase of robotic adoption. Our findings suggest that robotic surgery offers potential advantages, particularly in terms of lower conversion rates to open surgery and shorter postoperative hospital stays, even when performed during the early stages of the learning curve. However, given the substantial costs associated with robotic platforms and the limited evidence on long-term benefits, it remains essential to critically assess the overall value of robotic surgery compared to the more cost-effective laparoscopic approach.

Future research should aim to address the current evidence gaps by focusing on long-term oncological outcomes, cost-effectiveness analyses, functional recovery, and patient-reported experiences. Additionally, efforts to standardize robotic surgery training and credentialing across institutions will be crucial to ensure consistent surgical quality and optimize patient outcomes as the adoption of robotic technologies continues to expand in colorectal surgery.
